# Glycine-rich RNA-binding cofactor RZ1AL is associated with tomato ripening and development

**DOI:** 10.1093/hr/uhac134

**Published:** 2022-08-02

**Authors:** Xindi Li, Yongfang Yang, Ni Zeng, Guiqin Qu, Daqi Fu, Benzhong Zhu, Yunbo Luo, Oren Ostersetzer-Biran, Hongliang Zhu

**Affiliations:** College of Food Science & Nutritional Engineering, China Agricultural University, Beijing 100083, China; Department of Biochemistry and Biophysics, Texas A&M University, College Station, TX 77840, USA; Institute for Plant Genomics and Biotechnology, Texas A&M University, College Station, TX 77840, USA; Tsinghua-Peking Center for Life Sciences, School of Life Sciences, Tsinghua University, Beijing 100084, China; College of Food Science & Nutritional Engineering, China Agricultural University, Beijing 100083, China; College of Food Science & Nutritional Engineering, China Agricultural University, Beijing 100083, China; College of Food Science & Nutritional Engineering, China Agricultural University, Beijing 100083, China; College of Food Science & Nutritional Engineering, China Agricultural University, Beijing 100083, China; College of Food Science & Nutritional Engineering, China Agricultural University, Beijing 100083, China; Department of Plant and Environmental Sciences, Institute of Life Sciences, The Hebrew University of Jerusalem, Edmond J. Safra Campus - Givat Ram, Jerusalem 9190401, Israel; College of Food Science & Nutritional Engineering, China Agricultural University, Beijing 100083, China

## Abstract

Tomato ripening is a complex and dynamic process coordinated by many regulatory elements, including plant hormones, transcription factors, and numerous ripening-related RNAs and proteins. Although recent studies have shown that some RNA-binding proteins are involved in the regulation of the ripening process, understanding of how RNA-binding proteins affect fruit ripening is still limited. Here, we report the analysis of a glycine-rich RNA-binding protein, RZ1A-Like (RZ1AL), which plays an important role in tomato ripening, especially fruit coloring. To analyze the functions of *RZ1AL* in fruit development and ripening, we generated knockout *cr-rz1al* mutant lines via the CRISPR/Cas9 gene-editing system. Knockout of *RZ1AL* reduced fruit lycopene content and weight in the *cr-rz1al* mutant plants. *RZ1AL* encodes a nucleus-localized protein that is associated with Cajal-related bodies. RNA-seq data demonstrated that the expression levels of genes that encode several key enzymes associated with carotenoid biosynthesis and metabolism were notably downregulated in *cr-rz1al* fruits. Proteomic analysis revealed that the levels of various ribosomal subunit proteins were reduced. This could affect the translation of ripening-related proteins such as ZDS. Collectively, our findings demonstrate that *RZ1AL* may participate in the regulation of carotenoid biosynthesis and metabolism and affect tomato development and fruit ripening.

## Introduction

Tomato (*Solanum lycopersicum*) has been an optimal model plant for studying the ripening process of fleshy fruit for decades. During its ripening, there are physiological and metabolic changes accompanied by differential gene expression, including ethylene release, chlorophyll reduction, and carotenoid accumulation [1–3]. The dramatic increase in carotenoids in fruit contributes to the formation of fruit ripening color. Importantly, lycopene, an unoxygenated carotenoid (i.e. carotene), is a crucial contributor to tomato fruit pigmentation (red coloring) and normally accumulates to high levels during fruit ripening [4, 5]. Carotenoid metabolism has been described previously in tomato fruits [6, 7]. Geranylgeranyl diphosphate (GGPP), which is made of four molecules of isopentenyl pyrophosphate (IPP), serves as a precursor for many plastidial isoprenoids. Two molecules of GGPP are catalyzed by phytoene synthase (PSY) into a colorless 15-*cis*-phytoene pigment. In sequential reactions, catalyzed by phytoene desaturase (PDS), 15-*cis*-zeta-carotene isomerase (ZISO), ζ-carotene desaturase (ZDS), and carotenoid isomerase (CRTISO) in turn, phytoene undergoes desaturation and geometrical isomerization reactions into all-*trans*-lycopene. Cyclization of lycopene, by lycopene α-cyclase (LCYE) and lycopene β-cyclase (LCYB), leads to the production of α- and β-carotene, respectively. These can be further metabolized in the plastids into lutein or zeaxanthin. Over the last few decades, substantial progress has been made in studying the regulatory factors of carotenoid biosynthesis in ripening fruits, especially in tomato plants [8–11]. These included transcription factors, RNA editing activities, and non-coding RNAs (ncRNAs), which were shown to affect fruit development, ripening, and pigmentation. Here we explored the functions of the *RZ1AL* gene in tomato ripening and development.

RNA binding proteins (RBPs) play central roles in transcription, RNA metabolism, and translation, controlling gene expression programs in plants during embryogenesis, germination, growth, and development [12–17]. Many are characterized by canonical RNA-binding motifs, such as the RNA-recognition motif (RRM), zinc-finger, K-homology, and serine and arginine-rich (SR) domains [18]. RBPs may harbor additional domains that are required for protein–protein interactions, RNA/ligand specificity, or mediating post-transcriptional modifications [19–21]. These include glycine-rich regions, often arranged in domain repetitions, which are postulated to mediate protein–protein or protein–nucleic acid interactions [22].

Glycine-rich RNA-binding proteins (GRPs) are featured by a conserved RRM on the N-terminus and repetitive Gly-Gly-Arg (GGR) amino acid regions in the carboxy terminus [23–25]. GRPs are divided into five main subclasses [26, 27]. Among these is a group of proteins (i.e. Class IV GRPs) that are characterized by an internal (CCHC-type) zinc-finger motif, known as RZs [28]. The function of RZ proteins has been studied in model monocot and dicot plant species, including *Arabidopsis thaliana*, wheat (*Triticum aestivum*), and rice (*Oryza sativa*) [29–31]. In *Arabidopsis*, *AtRZ-1a*, *AtRZ-1b*, and *AtRZ-1c* were previously associated with enhanced cold tolerance [32, 33] and were also reported to affect seed germination under high salt and drought stresses [34]. *AtRZ-1a* was further associated with other types of abiotic stresses [29] and was shown to be ubiquitously expressed, generally acting as an RNA chaperone during cold adaptation [33, 34]. Likewise, *TaRZ2* in wheat and *OsRZ1* in rice are suggested to function as RNA chaperones, affecting the folding and stabilities of various RNAs *in vivo* [28, 30]. Recently, RNA-binding proteins such as *SlORRM4* have been reported to be involved in the regulation of tomato fruit ripening [8, 35]. However, the roles of RZ factors in the regulation of plant development and fruit ripening are limited. In this study, we analyzed the roles of an RZ-related protein (i.e. RZA1L) in tomato (*S. lycopersicum*), a key model plant for fruit ripening and one of the most important vegetable plants in the world [2]. The effects of knockout (CRISPR/Cas9) lines on the phenotype and fruit physiology in tomato during ripening are discussed.

## Results

### Phylogeny of the *RZ1AL* gene family in tomato

Previously, we used the SGN (SOL Genomics Network, https://solgenomics.net) to screen for putative ripening-related RNA binding proteins, which are homologous to known *Arabidopsis* RBPs. These led to the identification of several candidates, such as SlORRM4, which encodes a ripening-related mitochondrial RNA editing co-factor [35], as well as RZ-related genes encoding GRPs. RZs were first identified in tobacco (*Nicotiana sylvestris*) plants as an uncharacterized family of RNA-binding proteins containing an RNA-binding domain at the N-terminus, followed by a zinc finger (CCHC-type) motif and a glycine-rich C-terminal [36].

*Arabidopsis* harbors three RZ-related genes (*AtRZ1a*, *1c*, and *1c*), which act as RNA chaperones and play important roles under various stress conditions [18, 32, 33]. Using the tomato SGN database, we identified four genomic loci that display a high sequence homology to *RZ* genes in *A. thaliana*: the *SlRZ1A* (Solyc10g047130), *SlRZ1AL* (*SlRZ1A Like*, Solyc01g104840), *SlRZ1C* (Solyc11g008210), and *SlRZ1CL* (*SlRZ1C Like*, Solyc03g071560) genes ().

**Figure 1:**
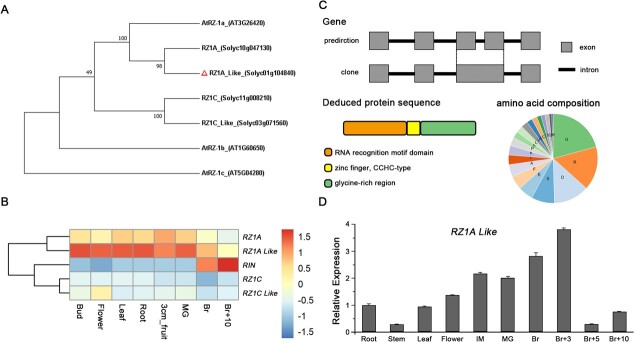
Analysis of RZ1AL phylogeny and gene expression. (A) Phylogenetic analysis of the RZ family in tomato and *Arabidopsis.* Genetic relatedness between seven RZ members was inferred from neighbor-joining tree-based evolutionary analyses with a bootstrap value of 1000. Numbers at the branches represent the percentage of replicate trees in which the associated taxa cluster together. (B) Heat map of relative gene expression of *RIN* and *RZs* in tomato in various tissues based on RNA-seq data. Relative gene expression of different tissues is normalized by corresponding expression levels of *RIN*. (C) Schematic diagram of the gene cloning and amino acid sequence of RZ1AL protein. (D) *SlRZ1AL* expression pattern in different tissues of tomato. Values are the mean ± standard deviation of three biological replicates.

Phylogenetic analysis suggested that two of the RZ paralogs in tomato, RZ1A and RZ1AL, are orthologous to AtRZ1a (Fig. 1A), with 68.75 and 60.99% identity in their amino acid sequences to *AtRZ1a*, respectively (Supplementary Data Table S1). The two related RZ1b and RZ1c proteins in *Arabidopsis* and the SlRZ1C and SlRZ1C-Like paralogs are clustered into two distinct branches (Fig. 1A), suggesting that these paralogs in *Arabidopsis* and tomato represent a gene duplication event in both species. Analysis of the expression profiles of the four *RZ* genes in tomato indicated that *RZ1AL* is expressed to relatively high levels in different tissues, including in fruits at the breaker (Br) stage (Fig. 1B), and to lower levels during later stages in fruit development and ripening (i.e. 10 days after the Br stage, Br + 10). *RZ1A* expression was also apparent in different tissues, but not in the fruits, while *RZ1C* and *RZ1C-like* were expressed to lower levels in the tissues tested. In comparison with the RZs in tomato, a high expression of *RIN* (*ripening inhibitor*) was specifically noted during the late-ripening stages (i.e. Br + 10) (Fig. 1B).

High-throughput sequencing (HTS) technology has replaced the more laborious construction and analysis of clone libraries but may also introduce errors to whole-genome sequencing (WGS) reads. Likewise, cloning of the *RZ1AL* gene from tomato indicated that the *RZ1AL* gene locus contains four exons and three introns, instead of the predicted five exons and four introns as annotated in the SGN (Fig. 1C). This was further supported by our RNA-seq analysis (PRJNA658729) of the tomato ‘Micro-Tom’ cultivar used in our study, as indicated in Supplementary Data Fig. S1. Additionally, the modified amino acid sequence of RZ1AL protein is provided in Supplementary Data Fig. S2. Notably, while both the original and modified sequences (Supplementary Data Fig. S2A) contained the RRM motif, the deduced protein sequence of the cloned *RZA1L* gene restored the conserved zinc-finger and GGR domains associated with plant RZ proteins that are missing in the original file (Supplementary Data Fig. S2B). Furthermore, sequence alignments of RZ1A proteins in model angiosperms (e.g. *Nicotiana tabacum*, *O. sativa*, *S. lycopersicum*, *Triticum aestivum*, and *Zea mays*) indicated that the C-terminal GGR motif is well conserved among other RZ1AL-related proteins in angiosperms (Supplementary Data Fig. S2C). We further analyzed the integrity of the reads in the upstream and downstream untranslated regions (UTRs) of the *RZ1AL* gene locus. Sequencing of different clones indicated the presence of two alternative 5′-UTRs for *RZ1AL* that are altered by 17 bases (Supplementary Data Fig. S3A), whereas the 3′-UTR was identified by a particular sequence of 315 nucleotides, following the TGA stop codon (Supplementary Data Fig. S3B).

The relative transcription level of *RZ1AL* in various tissues (i.e. roots, stems, leaves, and flowers) and during different stages in tomato fruit development and ripening [i.e. immature, mature green, Br, Br + 3, Br + 5, and Br + 10], was examined by RT–qPCR (Fig. 1D). Our data further indicated that RZ1AL is ubiquitously expressed in different tissues, with high expression levels seen in the roots and flowers, and more notably during early fruit ripening stages, peaking during the Br + 3 stage.

### *cr-rz1a* causes a ripening defect phenotype in tomato fruit

**Figure 2:**
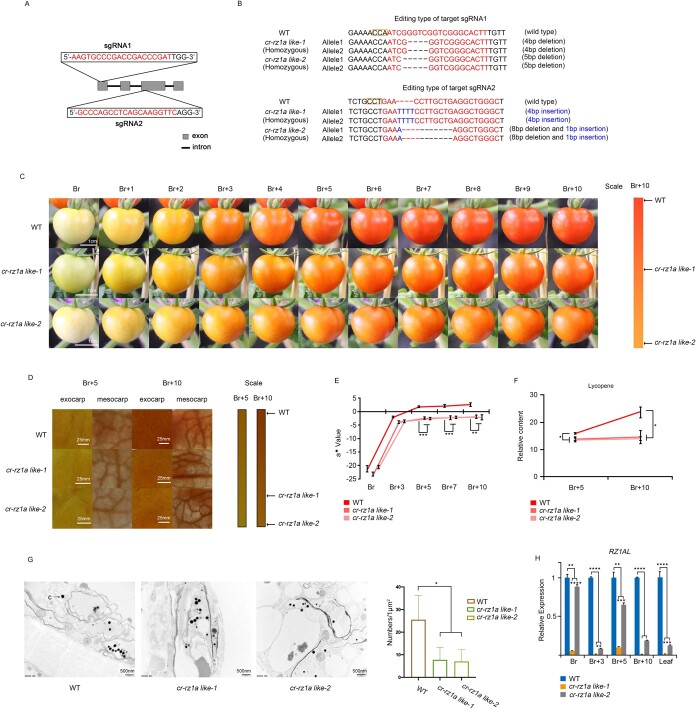
*cr-rz1al* mutant display reduced lycopene and altered fruit developmental phenotypes. (A) Two sgRNAs were designed to target the *RZ1AL* gene. The sgRNA sequence is colored red. (B) Genotyping of *cr-rz1al* mutants established by the CRISPR/Cas9 editing system. The sgRNA sequences are colored red, while PAM (protospacer adjacent motif) is marked by orange boxes. (C) Fruit ripening in *cr-rz1al* and WT*.* Scale bars represent 1 cm. (D) Microscopic analyses of the exocarp and mesocarp of WT and *cr-rz1al* mutant. Scale bar, 25 mm. (E) The a^*^ values mean chromaticity on a green (−) to red (+) axis. ^**^P < .01, ^***^P < .001. (F) Accumulation of lycopene in fruits of WT and *cr-rz1al* mutant at Br + 5 and Br + 10 stages. Values are means ± standard deviation of three replicates. ^*^P < .05. (G) TEM image of epidermal cells and statistical analysis of numbers of carotenoid-containing structures in WT and *cr-rz1al* fruits at Br + 10. c, carotenoid-containing structures. Scale bar, 500 nm. ^*^P < .05. (H) Gene expression analysis of *RZ1AL* in WT and *cr-rz1al* by RT–qPCR. Expression of *RZ1AL* in WT at different tissue and ripening stages (Br, Br + 3, Br + 5, Br + 7, Br + 10, and leaf) was considered to be 1 to show the relative expression of edited mRNA of *RZ1AL* at corresponding tissue and ripening stages. The decrease in *RZ1AL* gene expression in *cr-rz1al* mutants was due to nonsense-mediated mRNA decay.

To analyze the putative roles of RZ1AL in fruit development and ripening, we established several independent *cr-rz1al* mutant lines mediated by CRISPR/Cas9 genome editing. For this purpose, two single-guide RNAs, termed sgRNA1 and sgRNA2, were designed to target the first and third exons of the *RZ1AL* gene, using the CRISPR-P server (http://cbi.hzau.edu.cn/cgi-bin/CRISPR) (Fig. 2A). *Agrobacterium*-mediated transformation of the CRISPR/Cas9 sgRNAs vectors yielded 13 *T*_0_ independent transgenic lines, six of which were confirmed as genome-edited (i.e. heterozygous, biallelic, and chimeric mutant lines) (Supplementary Data Fig. S4).

We generated homogeneous mutants with two distinct genotypes of the *RZ1AL* locus in the *T*_1_ regeneration of *cr-rz1al* plants to further explore the function of *RZ1AL* in tomato development and fruit ripening. One of the mutant lines (here termed *cr-rz1al-1*) had a 4-bp deletion at the sgRNA1 and a 4-bp insertion at sgRNA2, while another mutant line (i.e. *cr-rz 1al-2*) carried a 5-bp deletion at sgRNA1 and an 8-bp deletion combined with 1-bp insertion at sgRNA2 (Fig. 2B). No off-target mutations were detected by PCR combined with Sanger sequencing, suggesting that sgRNA1 and sgRNA2 are specific for their respective recognition sites (Supplementary Data Table S2, Supplementary Data Fig. S5).

Both *cr-rz1al-1* and *cr-rz1al-2* displayed altered fruit ripening phenotypes (Fig. 2C). For the early stages of fruit ripening, no obvious differences in coloring were seen, i.e. from the Br stage until Br + 3 (Fig. 2C). But starting at the Br + 5 stage both mutant lines (*cr-rz1al-1* and *cr-rz1al-2*) exhibited delayed ripening phenotypes. The peel colors of the fruits of *cr-rz1al-1* and *cr-rz1al-2* seemed paler than those of the wild-type (WT) plants at the Br + 5 stage, which gradually turned orange and then red (Fig. 2C). While the fruits of WT plants were fully red at the Br + 10 stage, the fruits of *cr-rz1al* mutants remained orange in color. This was further apparent in the colors of the exocarp and mesocarp of fruits collected from the mutants or WT plants at the Br + 5 and Br + 10 stages (Fig. 2D). The pericarp color was measured with a colorimeter, utilizing the CIE L^*^a^*^b color system [37]. In this assay, the a^*^ value refers to the position between red and green, as defined by the reduction in chlorophyll and the accumulation of carotenoids, such as β-carotene and lycopene, producing the distinguishing yellow and red colorations, respectively [38]. The a^*^ (redness) values of both mutant lines were significantly reduced compared with the WT plants, at different stages throughout fruit ripening (Fig. 2E). In contrast, these assays suggested that there were no significant differences in the b^*^ and L^*^ values between the mutants and WT plants (Supplementary Data Fig. S6).

Differences in fruit ripening between the WT and *cr-rz1al* mutant plants were also described by quantifying the accumulation of different pigments at Br + 5 and Br + 10 stages (Fig. 2F). These assays indicated that lycopene content in WT fruit increased sharply from stage Br + 5 to stage Br + 10, whereas the carotene levels remained low in the mutants (i.e. the lycopene concentrations in both *cr-rz1al* lines were ~30% lower than in WT fruit at the Br + 10 stage; Fig. 2F). While lycopene was reduced in the fruits of the mutants, other carotenoids, such as phytoene, ζ-carotene, phytofluene, β-carotene, and lutein, were rather found to accumulate to higher levels in *cr-rz1al* mutants (Supplementary Data Fig. S7). Transmission electron microscopy (TEM) indicated a significantly reduced number of osmiophilic globules (carotenoid-containing structures) in *cr-rz1al* at the Br + 10 stage, compared with the WT fruits (Fig. 2G). The gross weight of individual fruits in WT plants was ~30% higher than that of the *cr-rz1al* mutants (Supplementary Data Fig. S8A), which in turn showed comparable color turning (Supplementary Data Fig. S8B) and similar respiratory rates (Supplementary Data Fig. S9A) and ethylene levels (Supplementary Data Fig. S9B) to those of the WT plants.

We considered that the above effects are tightly correlated with the loss of *RZ1AL*. This was strongly supported by RT–qPCR analyses that indicated that the accumulation of *RZ1AL* transcripts at the Br + 10 stage was ~80% lower in *cr-rz1al-2* and >90% lower in the *cr-rz1al-1* mutant compared with the WT plants (Fig. 2H). The decrease in *RZ1AL* gene expression in *cr-rz1al* mutants was due to the formation of a premature termination codon in the edited transcript before the original termination codon, resulting in nonsense-mediated mRNA decay [39]. These data coincide with the mutations produced in the *RZ1AL* gene locus by CRISPR/Cas9 genome editing, and further suggested that the loss of RZ1AL function(s) cannot be compensated by the other three RZ-related proteins in tomato (Fig. 1). The ripening defect seen in the fruits of *cr-rz1al* mutants cannot be directly attributed to altered respiratory functions or ethylene production (Supplementary Data Fig. S9).

### The *RZ1AL* gene encodes a nuclear-localized RNA-binding protein

**Figure 3:**
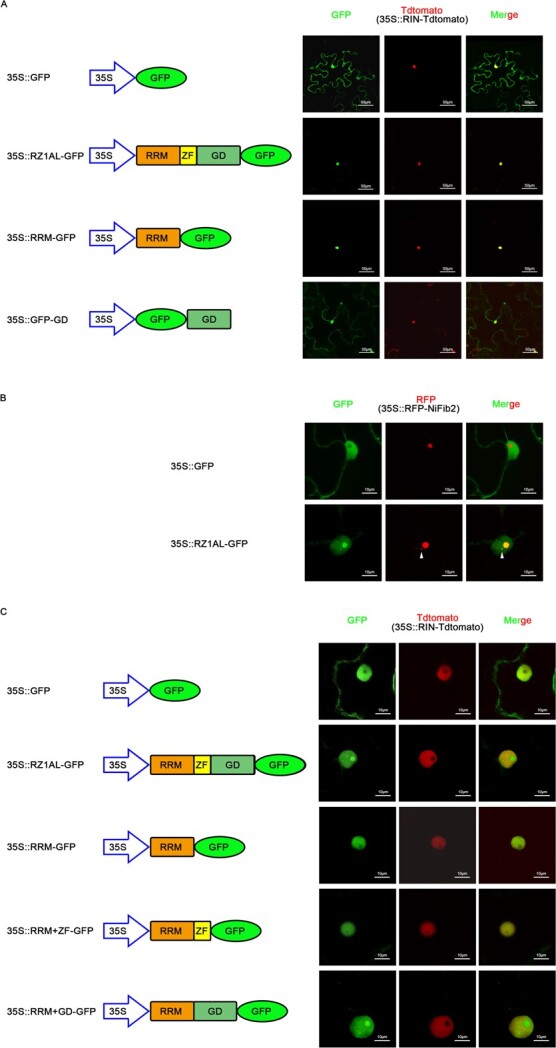
RZ1AL-GFP is located in the nucleus and associated with Cajal bodies. (A) Nuclear localization of RZ1AL. Tobacco leaves were used for detecting the fluorescence signal. 35S::GFP acted as a control and 3S::RIN-Tdtomato was used as a nuclear marker. Scale bar, 50 μm. (B) Nucleolus localization of RZ1AL. 35S::RFP-NiFib2 was used as a nucleolus marker. Scale bars, 10 μm. (C) Nuclear and nucleolus localization of different domains. Scale bars, 10 μm.

The intracellular location of RZ1AL was examined by green fluorescent protein (GFP) localization analyses in tobacco (*N. tabacum*) plants. Based on the distribution of the GFP signals, we considered that RZ1AL is localized within the nucleus (Fig. 3A). This was evident by the co-localization of RZ1AL-GFP with the transcription factor *RIN* fused to the Tdtomato marker (RIN-Tdtomato) [40]. Within the nucleus, RZ1AL-GFP showed a diffuse distribution pattern, with the protein concentrating in small spherical nuclear bodies (Fig. 3B). Co-localization analyses with red fluorescent protein (RFP)-NbFib2 [41], strongly suggested that RZ1AL-GFP is found in Cajal bodies, subnuclear structures that consist largely of protein and RNA. We also analyzed the intracellular locations of several truncated *RZ1AL* gene constructs lacking the glycine-rich domain and zinc finger domain, or the RRM and zinc finger domains (Fig. 3A and C). While the RRM region alone was sufficient to promote a nuclear localization (Fig. 3A and C), the presence of both the glycine-rich domain and RRM motifs promoted a Cajal body localization, as indicated by their co-localization with the NbFib2 marker [41].

### *RZ1AL* affects the expression level of several key enzymes in the carotenoid biosynthetic pathway

**Figure 4:**
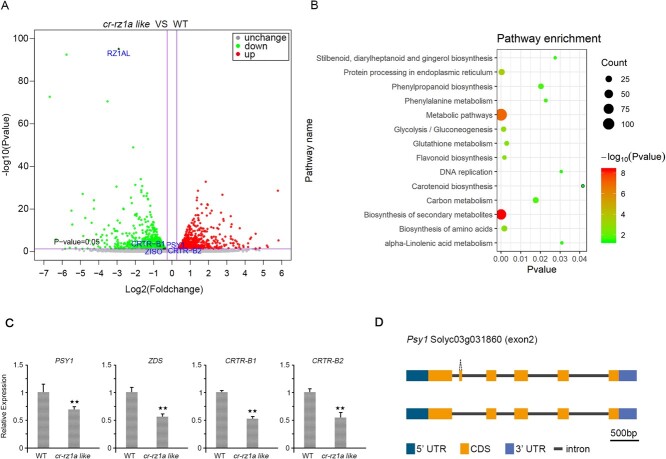
Global gene expression analysis, using RNA-seq, indicates a role for RZ1AL in the expression of carotenoid-related genes during fruit ripening. (A) Volcano diagrams of DEGs in *cr-rz1al* fruits compared with WT at the Br + 10 stage. *P*-value <0.05 indicates that DEGs are statistically significant. Downregulated and upregulated DEGs are colored green and red, respectively. Several key genes, including *RZ1AL*, *PSY1*, *ZISO*, *CRTR-B1*, and *CRTR-B2*, are highlighted with blue dots. (B) GO functional enrichment analysis of downregulated DEGs between *cr-rz1al* and WT plants. (C) RT–qPCR of key genes associated with the carotenoid pathway in *cr-rz1al* and WT plants. ^**^P < .01. (D) Schematic diagram of alternative splicing events of the *PSY1* gene in *cr-rz1al* versus WT plants.

To analyze the gene-function(s) of RZ1AL in tomato fruit ripening and coloring, we compared the transcriptome landscapes (i.e. RNA-seq analyses; PRJNA658729) of knockout *cr-rz1al* with those of WT plants in tomato fruits at the Br + 10 stage (Supplementary Data Fig. S10A). The RNA-seq data obtained from three individual WT plants and three *cr-rz1a-1* plants indicated specific changes in the gene expression patterns between the control and the mutant plants (Supplementary Data Fig. S10B). Overall, 1733 differentially expressed genes (DEGs) were seen between the *cr-rz1a-1* and WT fruits, including 867 genes that were downregulated and 916 genes that were upregulated in the mutants in comparison with the WT plants (Supplementary Data Fig. S9C). Notably, among the most affected transcripts that were downregulated in the mutants were four key genes associated with carotenoid metabolism and biosynthesis, i.e. *CRTR-B1* (Solyc06g036260), *CRTR-B2* (Solyc03g007960), *PSY1* (Solyc03g031860), and *ZISO* (Solyc12g098710) (Fig. 4A). Gene Ontogeny (GO) enrichment analysis showed that downregulated genes in *cr-rz1al* were enriched in some ripening-related pathways, including the carotenoid biosynthesis pathway, flavonoid biosynthesis pathway, and metabolic pathway (Fig. 4B). RT–qPCR analyses supported the integrity of the RNA-seq data and further indicated that *CRTR-B1*, *CRTR-B2*, *PSY1*, and *ZDS* are indeed expressed to lower levels in the mutant (Fig. 4C).

**Figure 5:**
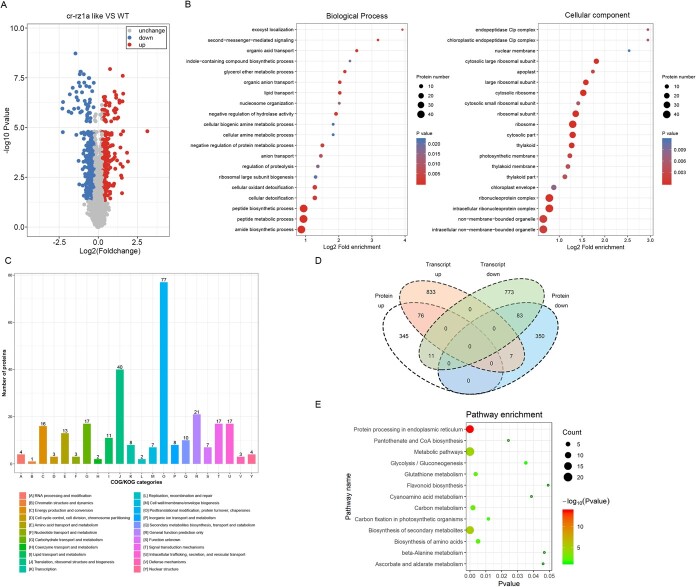
Proteomic analysis of WT and *cr-rz1al* mutant plants shows global changes in protein expression. (A) Volcano map of differentially expressed proteins between *cr-rz1al* and WT. Downregulated and upregulated proteins in *cr-rz1al* are colored blue and red. (B) GO functional enrichment analysis of downregulated expression proteins between *cr-rz1al* and WT. (C) COG/KOG category analysis of downregulated expression proteins between *cr-rz1al* and WT. (D) Integrated analysis combining RNA-seq with proteomics in *cr-rz1al*. Fold change >1.2, *P* < .05. (E) GO enrichment analysis for overlap between downregulated transcripts and downregulated proteins in *cr-rz1al*.

As some RZ members were previously shown to affect the processing (splicing or alternative splicing) of various genes, plausibly by interacting with various splicing co-factors in the nucleus [42], we used the Majorbio online server (https://cloud.majorbio.com/) to address potential differences in the splicing activities of nuclear genes between *cr-rz1al* and WT plants. As indicated in Supplementary Data Table S3, differences (*P* < .05) in the splicing patterns between *cr-rz1al* mutant and WT plants included exon skipping, intron retention, and altered splicing of introns within different 5′- or 3′-UTRs. These also included variations in the splicing of exons (i.e. exon skipping) in *PSY1*, which encodes a key enzyme in the carotenoid biosynthesis pathway (Fig. 4D). All in all, these data suggest that the loss of *RZ1AL* affects the expression level of many different genes, including those required for carotenoid biosynthesis in tomato fruits during ripening.

### Integrating proteomics and transcriptomics for systematic analysis of roles of *RZ1AL* in regulating tomato fruit ripening

Following the demonstration that RZ1AL is associated with the Cajal bodies within the nucleus (Fig. 3B) and affects the expression of various genes in tomato fruits (Fig. 4, Supplementary Data Fig. S10), we also addressed the possibility of global changes in protein expression between *cr-rz1al* knockout mutants and WT plants (Supplementary Data Fig. S11). The proteomic data indicated 39 351 peptides in total, with 35 650 of the sequences identified as unique peptides that corresponded to 6610 different proteins, of which 5542 were quantifiable (Supplementary Data Fig. S11A). The Pearson correlation coefficient for these data was used to evaluate protein quantification repeatability (Supplementary Data Fig. S11B). From the set of quantifiable proteins, 872 were identified with a fold change of >1.2 (*P* < 0.05); 432 of the proteins were increased and 440 were reduced in the mutant (Fig. 5A, Supplementary Data Fig. S11C).

Next, we performed an analysis of proteomic changes based on the clustering of orthologous groups (COGs; http://www.ncbi.nlm.nih.gov/COG/) and compared these databases with the transcriptomic analyses. These data seemed to coincide with the fold-enrichment of various proteins in the *cr-rz1al* mutant, compared with the WT plants (Fig. 5B). An overview of different proteins clustered into orthologous groups whose accumulations changed between the WT and *cr-rz1al* mutant plants is provided in Fig. 5C. Notably, the largest cluster (i.e. 77 different proteins) contains enzymes associated with post-translational modifications, protein turnover, and chaperones, while the second-largest cluster (i.e. 40 different proteins) is linked to ribosome assembly or biogenesis and post-translational modifications (Fig. 5C). Enzymes associated with amino acid transport, metabolism, protein translation or ribosome functions, and biosynthesis were generally downregulated in the mutant. Decreased steady-state levels were also noted in the cases of various ribosomal subunits, such as L2, L3, and L13 (Supplementary Data Fig. S12). Likewise, the levels of proteases (such as Clp) and various RNA-binding proteins found in ribonucleoprotein complexes were generally reduced compared with the WT plants.

Integration of the proteomic and transcriptomic data is indicated in Fig. 5D and Supplementary Data Table S4. Of the different genes that showed altered expression in *cr-rz1al* mutants, 176 were also identified by the proteomic analyses. These included 93 genes whose transcripts were found to be reduced in the mutant, the majority of which (81) also demonstrated lower (1.25- to 5-fold) protein levels (Supplementary Data Table S4). These included *ZISO* and a gene encoding the 60S ribosomal protein L3, as well as various other genes associated with translation, post-translational processing, and biosynthesis of amino acids (Fig. 5E). Of the 83 genes whose mRNA levels were upregulated in *cr-rz1al*, 76 showed higher protein levels in the mutant. These data indicate that the loss of *RZ1AL* disrupts the expression profile of many genes associated with cellular metabolism, carotenoid biosynthesis, and translation apparatus during early fruit ripening.

## Discussion

The maturation of fruits is a sophisticated developmental process that necessitates highly coordinated gene and protein expression [2, 43]. RNA binding proteins seem to play essential roles in fruit development and ripening by controlling the gene expression profiles required in the initiation of plant development, seed maturation, berry enlargement, coloring, and ripening. Here we report the analysis of a glycine-rich RNA-binding factor, *RZ1AL*, the functions of which are required during fruit ripening and development in tomato plants.

There is a series of physiological and metabolic changes during fruit ripening, including fruit color change, volume increase, sugar, and acid ratio change, and ethylene release [2]. In particular, the red color on the fruit surface has attracted much attention because it is an important indicator to evaluate the maturity and quality of tomato fruits [44]. In tomato, carotenoids, mainly consisting of lycopene, are the important contributors to the red color of ripening fruit. *In vivo*, lycopene and other carotenoids were biosynthesized in the carotenoid pathway coordinated by some enzymes with catalytic activity, like *PSY*, *PDS*, and *ZDS* [6]. RNA-seq analyses revealed that the expression of many genes is either up- or down-regulated in the fruits of *cr-rz1al* mutants. These include reduced mRNA or protein levels of key enzymes of the carotenoid biosynthesis pathway (Fig. 4, Supplementary Data Fig. S10, Supplementary Data Tables S3 and S4), resulting in reduced lycopene in *cr-rz1al* at Br + 10. This means that it is impossible for *cr-rz1al* fruit to gain the same red color as WT fruits during ripening because reduced lycopene is the most abundant carotenoid in mature tomato fruits. Notably, the presence of feedback regulation in the carotenoid biosynthesis and metabolism pathway makes the changes of other carotenoids during fruit ripening more complicated in the *cr-rz1al* mutant [6]. Based on Supplementary Data Fig. S7, in the Br + 5 stage the contents of various kinds of carotenoids including phytoene in *cr-rz1al* mutants were higher than those in WT (phytoene, phytofluene, ζ-carotene). This suggests that lycopene synthesis is blocked because of the accumulation of upstream carotene (the substrate required for lycopene synthesis). However, with the progress of ripening, only the lycopene content decreased significantly, and other carotenoid contents gradually returned to the WT level. This suggested that the feedback regulation [6] in the pathway affected the content of carotenoids upstream of lycopene, which means that the reduced lycopene in the early stage transmitted the signal to the upstream carotenoids, thus promoting the accumulation of carotenoids upstream in the late stage. Molecular and physiological analyses of knockout *RZ1AL* mutant lines (*cr-rz1al*) established by the CRISPR/Cas9 editing system (Fig. 2) indicated altered fruit ripening, with reduced lycopene content.

The RNA-seq data also indicated altered plastidial gene expression, where the proteolytic Clp machinery was notably downregulated in the mutant (Fig. 5C). One of the most critical developmental stages associated with fruit ripening is the transformation of chloroplasts into chromoplasts. During this process, the chromoplast becomes enriched with osmiophilic globules that contain high levels of carotenoids, mainly lycopene and β-carotene [45]. Microscopic analyses indicated that the number of osmiophilic globules is significantly reduced in *rz1al* mutants compared with the WT plants, especially at the late-ripening stage (i.e. Br + 10; Fig. 2G). These data seem to be closely related to the altered ripening phenotype of the *rz1al* fruits at the Br + 10 stage (Fig. 2C and D), whereas the fruit color and lycopene content are largely equivalent between the WT and mutant plants during the early stages of fruit development and ripening progress (Fig. 2E and F).

Increasing fruit size is an important part of tomato ripening and is a major concern for improving crop yield [46]. In general, it is thought that fruit size is directly related to the number of locules, suggesting that the greater the number of locules, the greater the size of the fruit [47–49]. In our data, we found that *RZ1AL* is important for tomato fruit size. In *cr-rz1al*, the weight of individual fruits is decreased significantly compared with WT. However, the number of locules is the same in *cr-rz1al* and WT (Supplementary Data Fig. S8A). It is difficult to explain the molecular mechanism of RZ1AL that regulates fruit size, and there are similar situations for other published mutants with a decreased fruit size and an unchanged locule number [50, 51]. *RZ1AL* could have a function in contributing biomass, but it still needs to be further studied.

Transcription factors, such as *RIN*, *NOR* (No-ripening), and *CNR* (colorless non-ripening), have been associated with tomato fruit development and ripening [52–54]. *RIN* is mainly associated with the progression of ripening, while it is unlikely to be necessary for ripening initiation [52, 55]. Based on published data and our results, neither *RIN* nor *RZ1AL* can affect the initial ripening stage of fruit, especially the color-turning stage from green to yellow. Both RIN and RZ1AL are localized to the nucleus, yet show different intranuclear locations, in which RZ1AL seems to be associated with the Cajal bodies (Fig.ure 3). In addition, differently from RIN, *RZ1AL* is slightly downregulated at later developmental stages (Br + 7 and Br + 10) (Fig. 1B). In summary, these results may indicate that the mechanism by which *RZ1AL* regulates tomato fruit ripening could be partially different from the *RIN* mechanism.

RNA polymerase II (Pol II) regulates the transcription of numerous mRNAs, as well as some small nuclear RNAs, in the genomes of eukaryotic organisms. Previously, it was shown that mRNA Pol II-driven transcription is associated with the Cajal bodies, a phenomenon that was also linked to RNA maturation and splicing [56, 57]. The altered gene expression and splicing activities we see in *cr-rz1al* may therefore relate to Pol II activities by a yet unknown mechanism. Similarly, reduced levels of various ribosomal subunits are expected to influence the translation of the mature transcripts in the cytoplasm of ripening tomato fruits. The altered expression and/or RNA metabolism defects in various ripening-related genes, such as *PSY1* and *ZISO*, are expected to result in reduced lycopene content in the fruits of *cr-rz1al*.

In summary, our data indicate a role for *RZ1AL* in fruit ripening and development. The *cr-rz1al* mutants display delayed fruit ripening, reduced coloring, and decreased weight, phenotypes that are closely associated with altered gene expression of ribosomal subunits and key enzymes of the carotenoid biosynthesis pathway. These results provide significant insights into the regulatory mechanisms of carotenogenesis during fruit development and ripening.

## Materials and methods

### Plant materials and growth conditions

WT tomato (*S. lycopersicum*, cv. ‘Micro-Tom’) plants were grown under standard growth conditions (i.e. 16 hours light/8 hours dark at 25°C) in a greenhouse. Leaf, stem, and root samples of 4-week-old WT plants and mutant line seedlings were collected. Flowers were labeled at anthesis to record fruit development progress, and berry tissues were collected at corresponding stages (i.e. immature, mature green, Br, Br + 3, Br + 5, and Br + 10), and immediately frozen in liquid nitrogen and stored at −80°C.

### pYLCRISPR/Cas9P_ubi-H_-RZ1AL vector construction and plant transformation

The CRISPR-P (http://cbi.hzau.edu.cn/crispr/) server was used for designing sgRNAs to target the *RZ1AL* gene locus in the tomato genome (Supplementary Data Table S5). The target sequences were selected to regions containing up to three consecutive T’s (to avoid their recognition by the RNA Pol III as a termination site), with a GC content >55%. As the secondary structure of sgRNAs may influence editing efficiencies, we used the MFOLD server (http://mfold.rna.albany.edu/) to confirm the integrity of the sgRNAs used in our assays. The sgRNAs were inserted into the binary plasmid pYLCRISPR/Cas9Pubi-H [58] and then the pYLCRISPR/Cas9P_ubi-H_-RZ1AL vector was transformed into ‘Micro-Tom’ plants by *Agrobacterium tumefaciens*-mediated transformation methods [6]. Putative transgenic lines were screened from hygromycin-resistant seedlings and confirmed by PCRs.

### DNA extraction and identification of genetically modified materials

For genomic DNA extraction from the WT and mutants, we used a DNAquick Plant System (Tiangen, China). The PCR program was as follows: 94°C for 3 minutes, 35 cycles of 94°C for 30 seconds, 55°C for 30 seconds, and 72°C for 30 seconds, followed by another single cycle of 72°C for 7 minutes. The PCR products were separated by agarose gel electrophoresis and sequenced to ensure their integrities. Superimposed sequence chromatograms representing the biallelic or heterozygous mutations were described using DSDecodeM (http://skl.scau.edu.cn/dsdecode/) combined with manual analyses. Specific oligonucleotides are listed in Supplementary Data Table S6.

### Analysis of the carotenoid content by LC–MS

Carotenoid extractions were performed generally as described previously [59]. Lyophilized tomato samples, obtained from fruits at the Br + 10 stage, were used for the extraction of pigments. Then, the pellet, dried by nitrogen blowing, was resuspended in 100 mL of ethyl acetate for each sample. We used the Accurate-MassHPLC1200/MS-QTOF 6520A (Agilent Technologies) system packed with a reverse-phase column, 4.6 × 150 mm, 3 μm (YMC) to identify and quantify the carotenoids. HPLC peak areas at 260–550 nm were integrated and calibrated, using external standards (e.g. α-carotene, β-carotene, and lycopene) at different ratios for the quantification of endogenous pigments in the fruits of WT plants and mutant lines. Carotenoids were quantified based on their established retention time and specific absorption spectrum [60, 61]. At least three independent extractions were performed for each mutant.

### Subcellular localization assays

The open reading frame (ORF) of RZ1AL was amplified by RT–PCR and cloned into a GFP expression vector using a one-step cloning kit (Vazyme). Likewise, the ORF of *RIN*, obtained by RT–PCR, was inserted into the tdTomato expression vector [62]. As controls, we used empty GFP and tdTomato vectors driven by the constitutive cauliflower mosaic virus 35S promoter. The fusion genes and vector controls were transferred to *A. tumefaciens* strain GV3101 and transiently expressed in tobacco leaves by *Agrobacterium*-mediated transformation. Forty-eight hours after infiltration the intracellular locations of the GFP and tdTomato signals were assayed by confocal microscopy (Olympus Nikon A1RMPSi).

### Transmission electron microscopy analyses

The internal structure and architecture of pericarps, obtained from WT and mutant lines at the Br + 10 stage, were studied by transmission electron microscopy (TEM) analyses, generally as described previously [6].

### RNA extraction and RT–qPCR analysis

Total RNA was extracted from tomato fruits using the Trizol reagent [63]. DNase treatment [64] was used to remove the DNA from the prepared total RNA sample. For cDNA synthesis, 2 μg DNA-free RNA preparations, were used in reverse transcription reactions with TransScript^®^ First-Strand cDNA Synthesis SuperMix (TransGen Biotech, Beijing, China). The relative expression of various transcripts in the mutant lines versus those in WT plants was determined by qPCR, using the cDNAs with SYBR Green PCR MasterMix and the CFX96 Touch Real-Time PCR Detection System (TransGen Biotech, Beijing, China). We performed at least three biological repeats for each sample, with three technical replicates in each assay, and the data were normalized with *ACTIN* (Solyc03g078400). Specific primers are listed in Supplementary Data Table S7.

### RNA-seq analysis

For RNA-seq, mRNA from total RNA was enriched by oligo-dTs coupled with magnetic beads, and then digested into fragments with a length of 300 bp (Novogene, China). The RNA-seq library was used in 150 bp pair-end sequencing using HiSeq PE150 (Illumina). All raw reads were uploaded to the NCBI Sequence Read Archive (http://www.ncbi.nlm.nih.gov/sra/; accession number PRJNA658729). For the heat map, the relative gene expressions of *RZ*s in tomato in various tissues were based on RNA-seq data and normalized by corresponding expression levels of *RIN*. The fold change was calculated as RPKM_cr-rz1al_/RPKM_WT_, where RPKM is reads per kilobase of transcript per million mapped reads. DEGs between *cr-rz1al* and WT were identified by the deseq2 package based on the threshold of |fold change| > 1.2 and a *P*-value of <.05.

### Proteomic analysis

Proteomic analyses were carried out by PTM-BIO (Zhejiang, China) as previously described [65]. Briefly, the extracted total proteins were digested into peptides with trypsin, as described in the users manual for the TMT kit/iTRAQ kit (ThermoFisher). Then, the peptides were quantitatively analyzed by LC–MS/MS. Finally, the resulting MS/MS data were analyzed using the Maxquant search engine (v.1.5.2.8). Differential expression proteins between *cr-rz1al* and WT were identified based on the threshold of |fold change| > 1.2 combined with a *P*-value of <.05. Four biological replicates were used here in proteomic analyses for WT and *cr-rz1al* mutants.

## Acknowledgements

This work was funded by grants to H.Z. from the National Natural Science Foundation of China (32061143022 and 31672208) and the 2115 Talent Development Program of China Agricultural University (1061-00109019), and by grants to O.O.B. from the Israeli Science Foundation (ISF; grants 1834/20 and 3254-2020). We greatly appreciate the provision of the binary vector pYLCRISPR/Cas9 system for us by Yaoguang Liu (South China Agriculture University). We also thank Chengguo Duan (Shanghai Center for Plant Stress Biology and Center of Excellence for Molecular *Plant Sci*ences) for providing the 35S:RFP-NiFib2 vector.

## Author contributions

X.L. and H.Z. designed the research. Y.Y. identified the *cr-rz1al* mutant by CRISPR/Cas9. X.L. conducted the experiments and prepared the manuscript. H.Z. and O.O.B. revised the manuscript. N.Z., G.Q., D.F., B.Z., and Y.L. provided the technology guide for the experiments.

## Data availability statement

All data related to this manuscript are available in this paper and its supporting materials.

## Conflict of interest

The authors state that the research was completed in the absence of any commercial or financial associations that could be construed as a possible conflict of interest.

## Supplementary data

Supplementary data is available at *Horticulture Research* online.

## Supplementary Material

Web_Material_uhac134
