# Supplementary material for: Glycine-rich RNA-binding cofactor RZ1AL is associated with tomato ripening and development
Source: Hortic Res. 2022 Aug 2;9:uhac134. doi: 10.1093/hr/uhac134 (PMC9350831; doi:10.1093/hr/uhac134)
Supplement: Web_Material_uhac134 [file web_material_uhac134.zip › Supplemental Table S1.docx]

**Table S1. The RZ protein family in Arabidopsis and tomato**

| *Arabidopsis thaliana* | *Solanum lycopersicum* | Identify% |
| --- | --- | --- |
| AT3G26420 (ATRZ-1a) | Solyc10g047130 (RZ1A) | 68.75 |
|  | Solyc01g104840 (RZ1A Like) | 60.99 |
| AT1G60650 (AtRZ-1b)  /AT5G04280 (AtRZ-1c) | Solyc03g071560 (RZ1C Like) | 46.79/51.64 |
|  | Solyc11g008210 (RZ1C) | 46.64/47.45 |
