# Supplementary material for: Glycine-rich RNA-binding cofactor RZ1AL is associated with tomato ripening and development
Source: Hortic Res. 2022 Aug 2;9:uhac134. doi: 10.1093/hr/uhac134 (PMC9350831; doi:10.1093/hr/uhac134)
Supplement: Web_Material_uhac134 [file web_material_uhac134.zip › Supplemental Table S2.docx]

**Table S2. Analysis of off-target sequences generated by the sgRNA1 and sgRNA2 in *cr-rz1al* mutants.**

| Off target | Sequence | Score | MMs | Locus | Gene | Region |
| --- | --- | --- | --- | --- | --- | --- |
| sgRNA1-offtarget1 | AAGTGCTCGCACGACCCGATGGG | 0.8 | 3MMs | SL2.50ch12:-46412381 |  | Intergenic |
| sgRNA1-offtarget2 | AGGTGCCCGCCCGATCCGCTGGG | 0.1 | 4MMs | SL2.50ch01:+69536769 |  | Intergenic |
| sgRNA1-offtarget3 | AGGTGCCCGCACGGCCCGATGGG | 0.1 | 4MMs | SL2.50ch05:+5455678 |  | Intergenic |
| sgRNA2-offtarget1 | **AT**CC**C**GC**A**TCAGCAAGGTTC**AGG** | 1.4 | 4MMs | SL2.50ch06:-36194089 | Solyc06g053420.2 | intron |
| sgRNA2-offtarget2 | GCCCAGCCTCA**A**CAA**A**GTTC**AGG** | 0.5 | 2MMs | SL2.50ch10:+39866806 | Solyc10g047130.1 | cds |
| sgRNA2-offtarget3 | GC**T**C**T**GCCTC**CT**CAAGGTTC**AAG** | 0.4 | 4MMs | SL2.50ch10:+61349140 | Solyc10g079880.1 | cds |
| sgRNA2-offtarget4 | GCCCA**AA**CT**T**AGCAAGG**G**TC**GGG** | 0.1 | 4MMs | SL2.50ch03:+68483666 | Solyc03g119970.2 | cds |

Red is used to label mismatched bases between off-target sequences and target sequences, and green represents PAM.
