# Supplementary material for: Glycine-rich RNA-binding cofactor RZ1AL is associated with tomato ripening and development
Source: Hortic Res. 2022 Aug 2;9:uhac134. doi: 10.1093/hr/uhac134 (PMC9350831; doi:10.1093/hr/uhac134)
Supplement: Web_Material_uhac134 [file web_material_uhac134.zip › Supplemental Table S5.docx]

**Table S5. Target sequences of the two pYLCRISPR/Cas9Pubi-H-RZ1AL (T1 and T2) vectors**

| sgRNA | Sequence | Score | GC (%) | Pairing with gRNA(bp) |
| --- | --- | --- | --- | --- |
| sgRNA1 | AAGTGCCCGACCGACCCGAT | 98 | 75 | 3 |
| sgRNA2 | GCCCAGCCTCAGCAAGGTTC | 96 | 75 | 5 |
