# Supplementary material for: Glycine-rich RNA-binding cofactor RZ1AL is associated with tomato ripening and development
Source: Hortic Res. 2022 Aug 2;9:uhac134. doi: 10.1093/hr/uhac134 (PMC9350831; doi:10.1093/hr/uhac134)
Supplement: Web_Material_uhac134 [file web_material_uhac134.zip › Supplemental Table S6.docx]

**Table S6. List of primers used in PCRs for the amplification of *RZ1AL* gene fragments.**

| Primer | Sequence |
| --- | --- |
| RZ1AL-T1-For | GTTAATCTAGGCCGATCTAATGAGTTAGCCC |
| RZ1AL-T1-Rev | GCCCCAAGACACCAATTGCCC |
| RZ1AL-T1-Seq-Rev | GAGAGAATTGTTTCAAGAAAAGAACATGGG |
| RZ1AL-T2-For | GATGCAAGAAAAAATGAGAATCTGAGTTCTTC |
| RZ1AL-T2-Rev | CACCAAAACATCTGCATCCAGCTAGG |
| RZ1AL-T2-Seq-Rev | GATAGCCTCCAGAACTGCGACGATC |
| RZ1AL-T1-offtarget1-For | GGAGAGACTGGGTGAAACAACTTCC |
| RZ1AL-T1-offtarget1-Rev | GGAGGGGGGTAATTTAATGAAAGGG |
| RZ1AL-T1-offtarget2-For | GCAGGGTCGCTGATGTTTCATAGG |
| RZ1AL-T1-offtarget2-Rev | CATAAGCTCCAAAGGAGGGGATTG |
| RZ1AL-T1-offtarget3-For | GACTCTTCTTTCCGCATGGTGGG |
| RZ1AL-T1-offtarget3-Rev | GGCAAAAGCTATATGAAAGTTGCGG |
| RZ1AL-T2-offtarget1-For | CACCATCCTCCTCAGACCCCAC |
| RZ1AL-T2-offtarget1-Rev | CAACAACTGAACGAGCGCAACTTC |
| RZ1AL-T2-offtarget2-For | GTTTGGTTTCTGATTTAGCCTGTGGC |
| RZ1AL-T2-offtarget2-Rev | CGCCTAGCTCTTCAGCTGCAACC |
| RZ1AL-T2-offtarget3-For | GCGTCCAGAAAGGGGCTTGG |
| RZ1AL-T2-offtarget3-Rev | GAGGGCAGTTTCTCCTCTGTGCC |
| RZ1AL-T2-offtarget4-For | GTCCAGATGGGTACTTGTCCGTCAC |
| RZ1AL-T2-offtarget4-Rev | GAAGAATCTTTTTCACACTCCGCCC |
| RZ1AL-T2-offtarget5-For | CGGACCCAGCCAGCTCAATTAC |
| RZ1AL-T2-offtarget5-Rev | GCTGCGAAGTGCGACAAAAGG |
