# Supplementary material for: Glycine-rich RNA-binding cofactor RZ1AL is associated with tomato ripening and development
Source: Hortic Res. 2022 Aug 2;9:uhac134. doi: 10.1093/hr/uhac134 (PMC9350831; doi:10.1093/hr/uhac134)
Supplement: Web_Material_uhac134 [file web_material_uhac134.zip › Supplemental Table S7.docx]

**Table S7. List of primers used in the RT-qPCRs**

| Primer | Sequence |
| --- | --- |
| RZ1AL-qPCR-For | GTTGATGCAAAGGTTGTACTTGAC |
| RZ1AL-qPCR-Rev | CCACCATAATCACGGCTACC |
| PSY1-qPCR-For | AGAGGTGGTGGAAAGCAA |
| PSY1-qPCR-Rev | TCTCGGGAGTCATTAGCAT |
| ZDS-qPCR-For | TGGAAGCATGTATGTTAGTGGG |
| ZDS-qPCR-Rev | CTTCAATTGCCTCGAACGCT |
| CRTR B1-qPCR-For | TAATCCTGGGTATCAAGTCGCT |
| CRTR B1-qPCR-Rev | ATCTCGCCAATCCATAAAAAGC |
| CRTR B2-qPCR-For | CAAACAACTATGGTGTATGGGA |
| CRTR B2-qPCR-Rev | CTCCACACACCTTTTGAGTAAC |
