# Supplementary figures and images for: Glycine-rich RNA-binding cofactor RZ1AL is associated with tomato ripening and development
Source: Hortic Res. 2022 Aug 2;9:uhac134. doi: 10.1093/hr/uhac134 (PMC9350831; doi:10.1093/hr/uhac134)

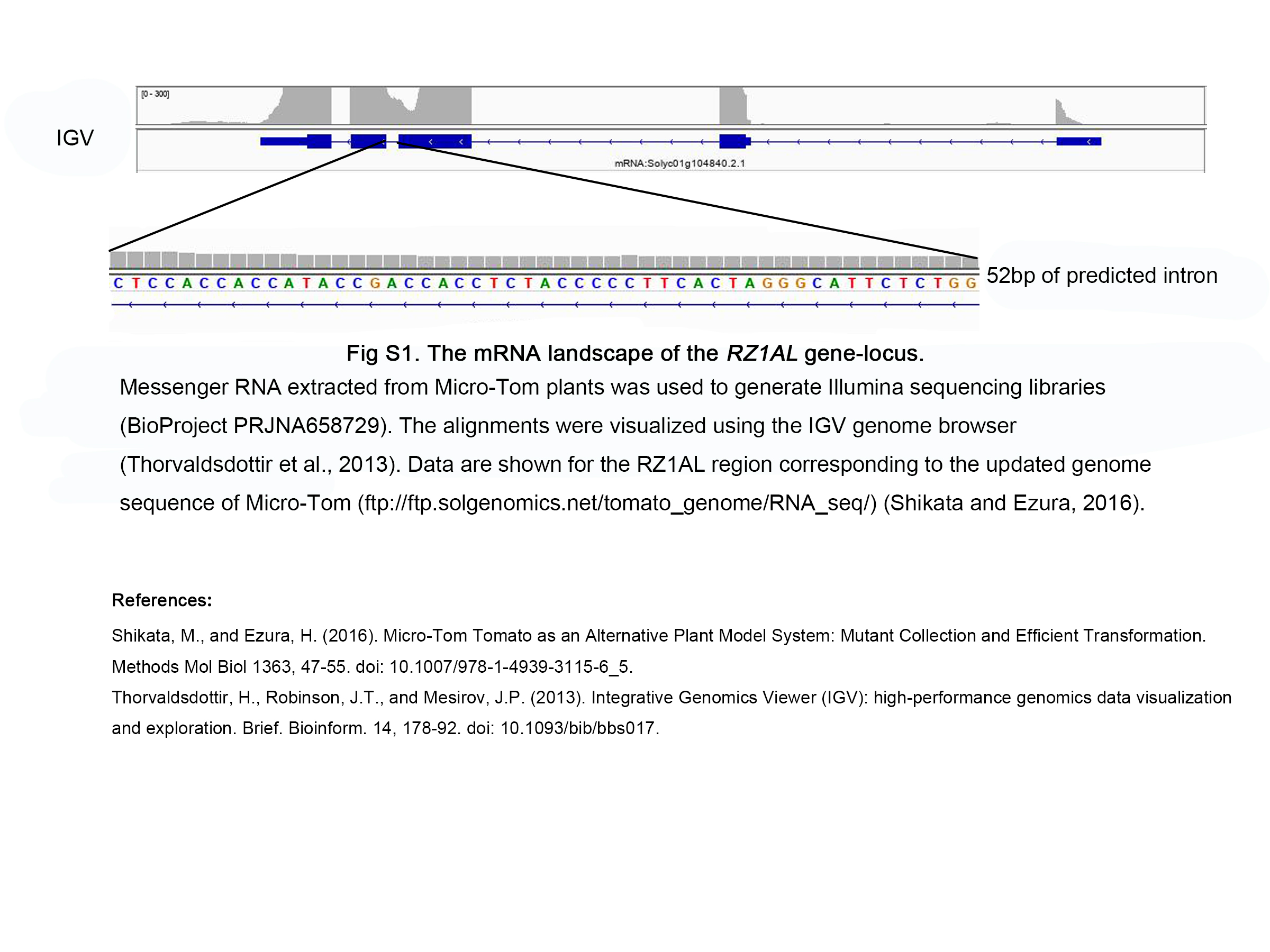

Supplement: Web_Material_uhac134 [file web_material_uhac134.zip › Supplement Fig. S1.jpg]

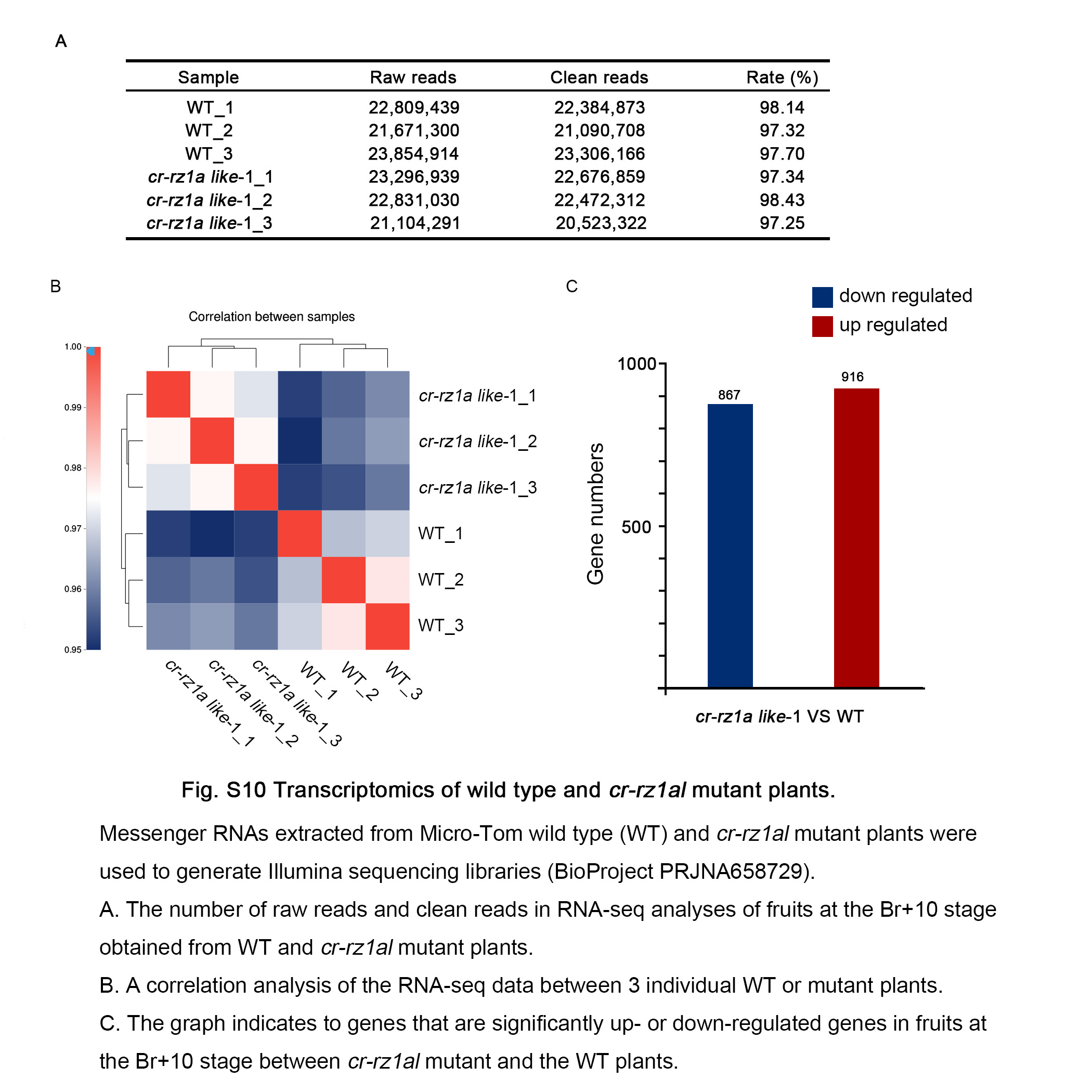

Supplement: Web_Material_uhac134 [file web_material_uhac134.zip › Supplement Fig. S10.jpg]

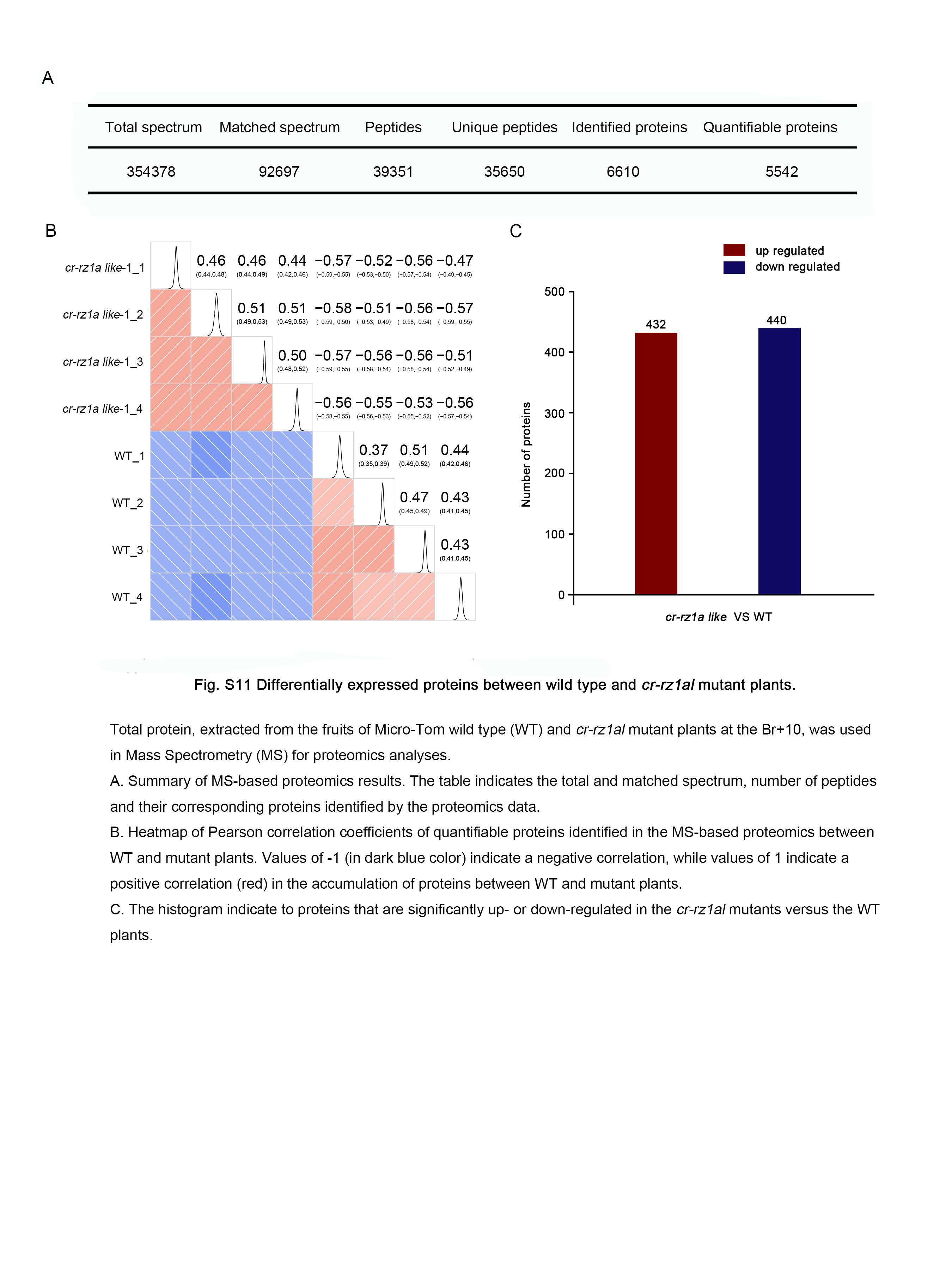

Supplement: Web_Material_uhac134 [file web_material_uhac134.zip › Supplement Fig. S11.jpg]

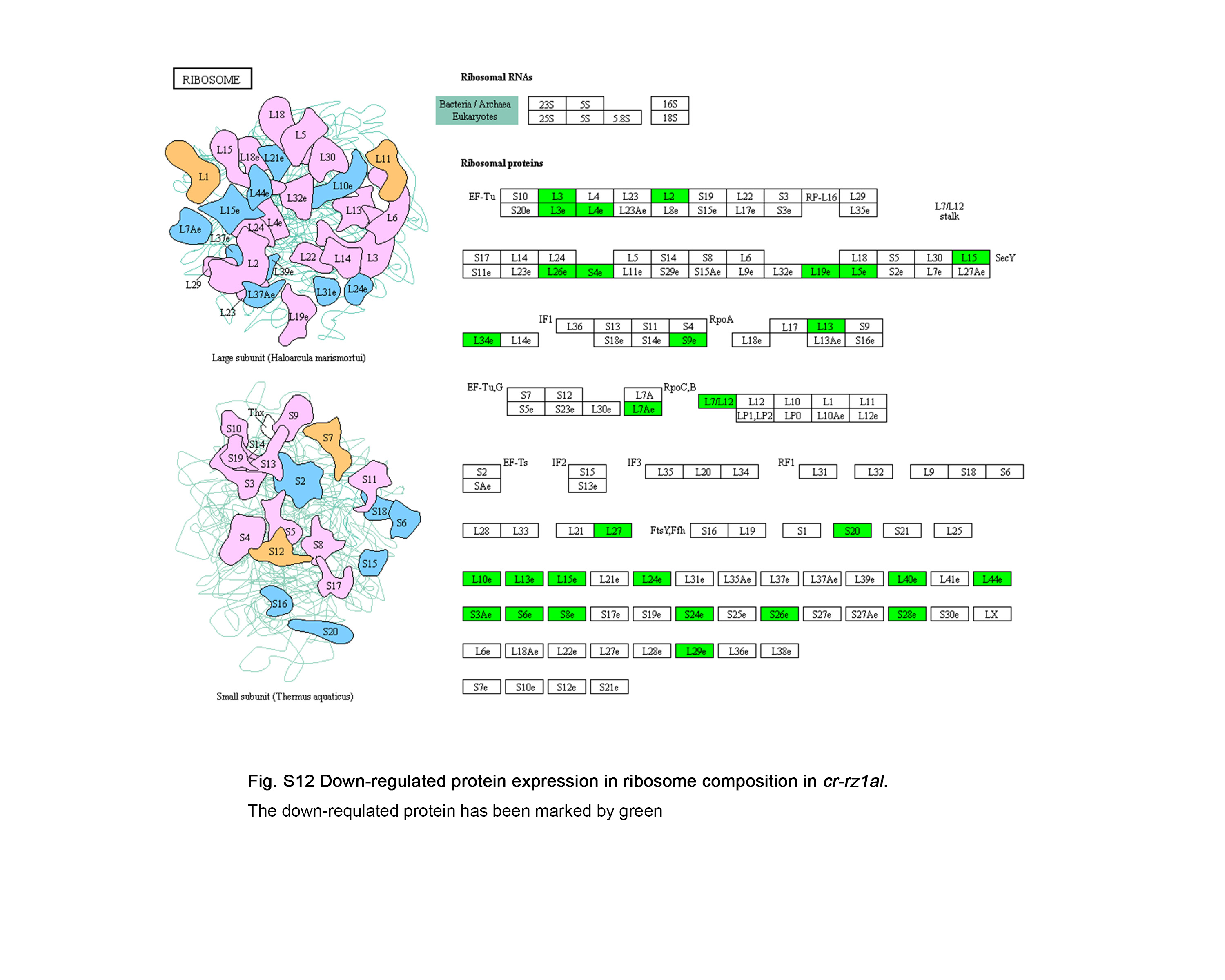

Supplement: Web_Material_uhac134 [file web_material_uhac134.zip › Supplement Fig. S12.jpg]

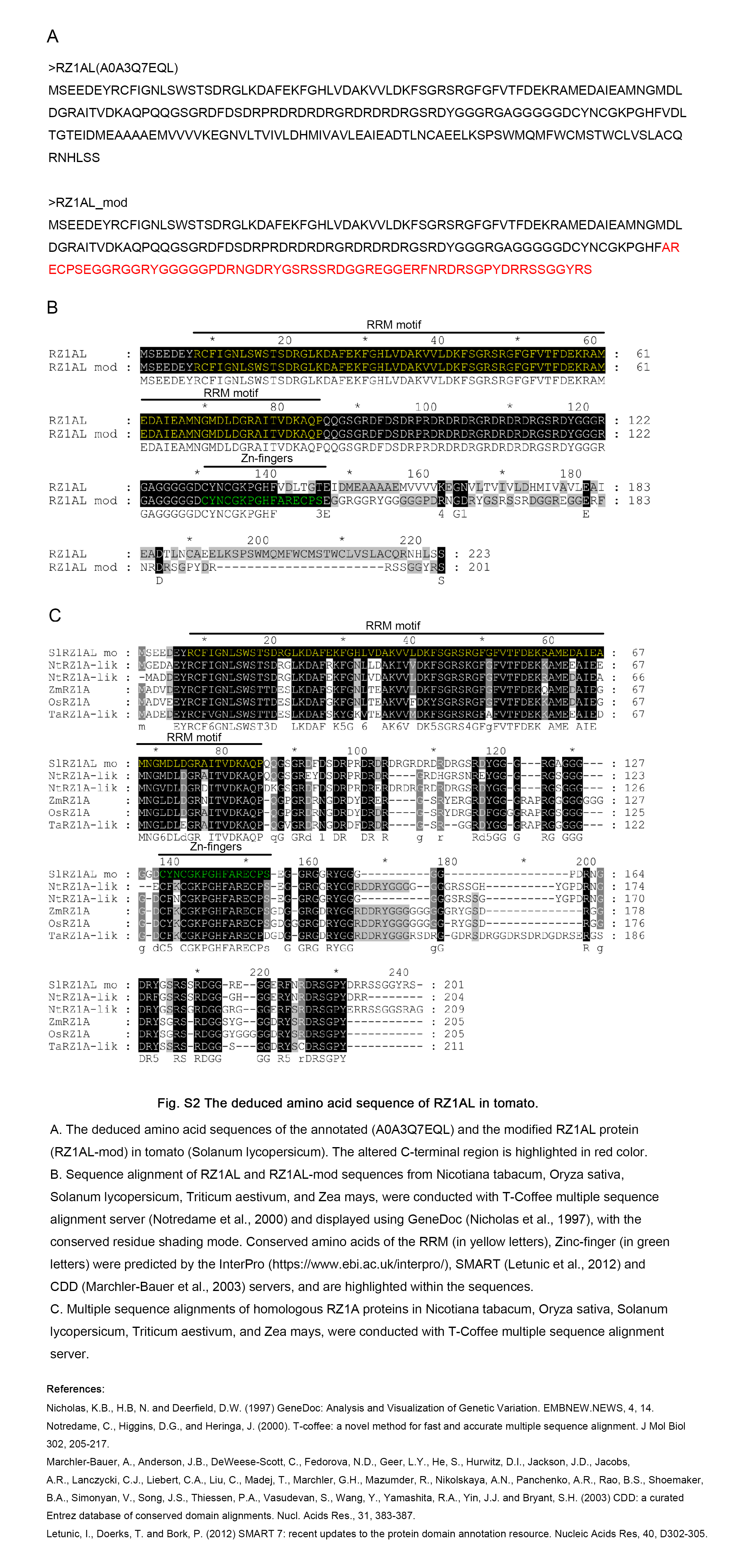

Supplement: Web_Material_uhac134 [file web_material_uhac134.zip › Supplement Fig. S2.jpg]

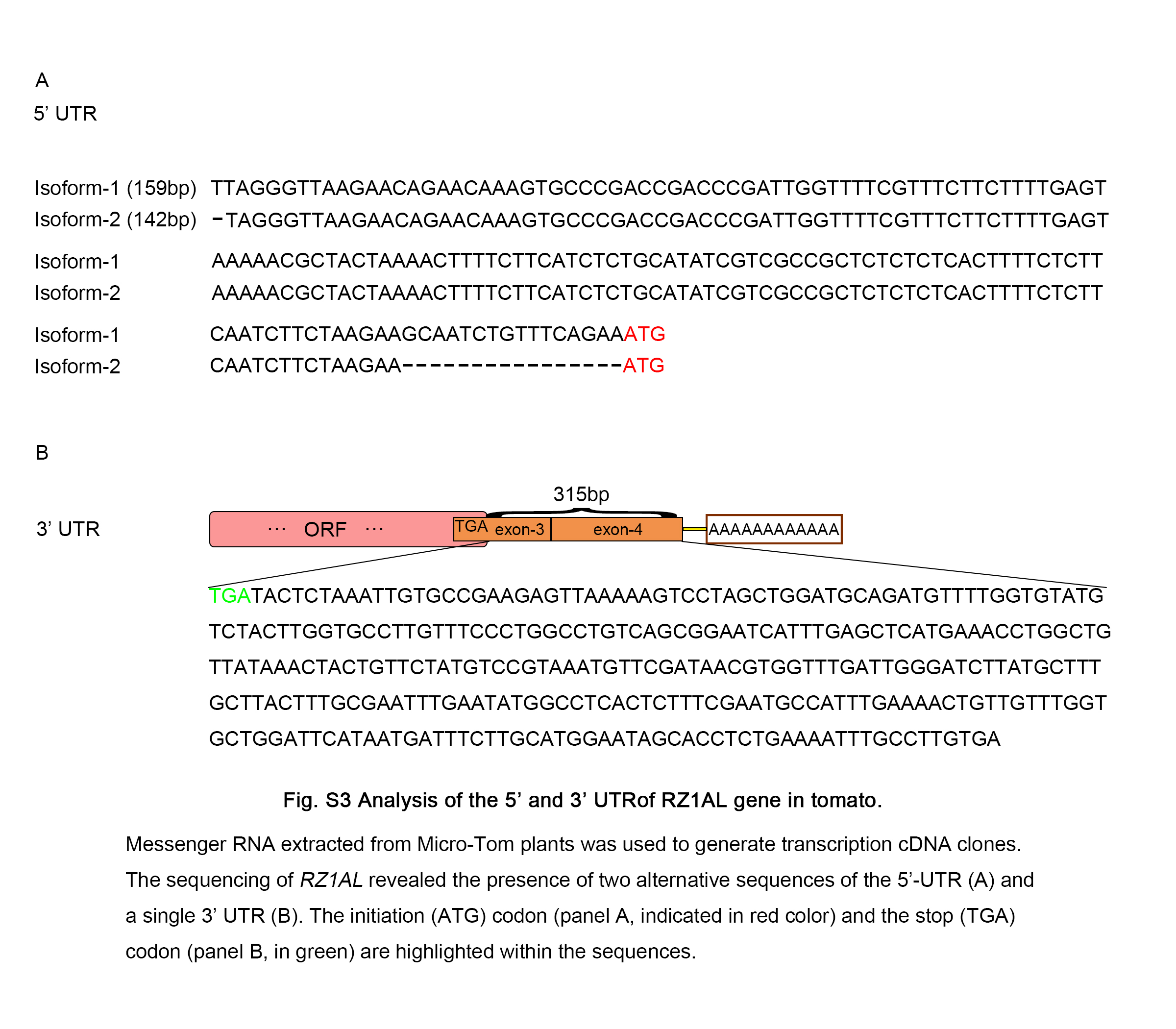

Supplement: Web_Material_uhac134 [file web_material_uhac134.zip › Supplement Fig. S3.jpg]

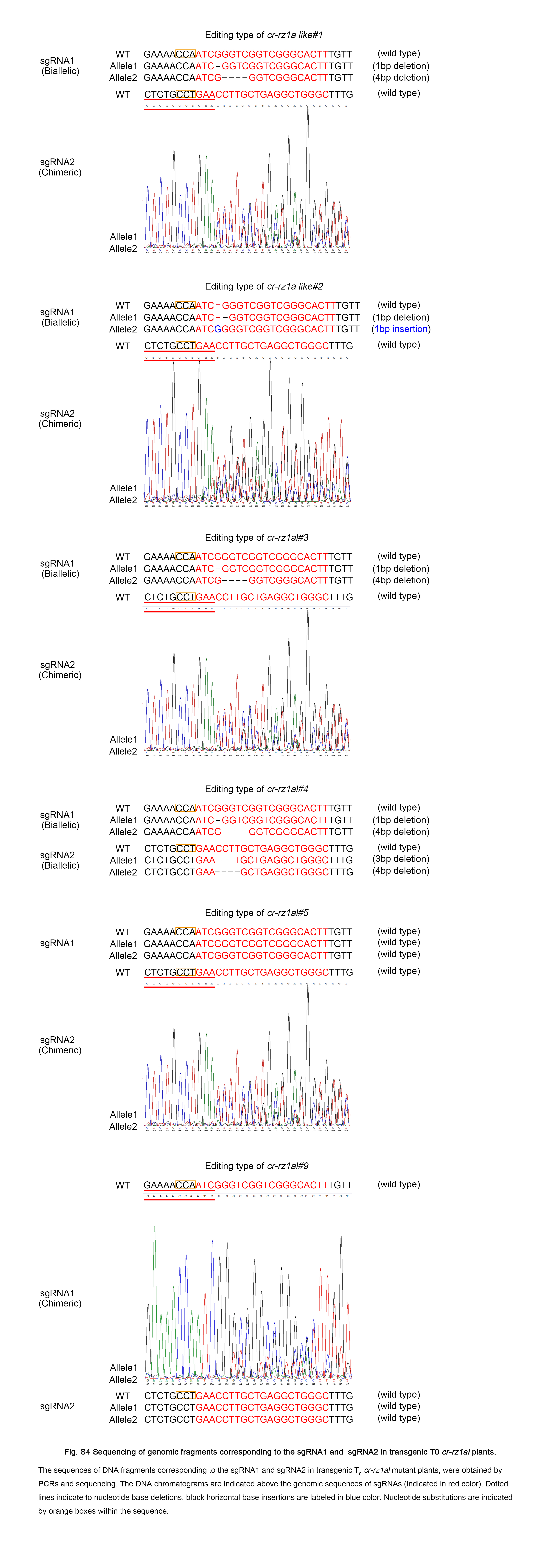

Supplement: Web_Material_uhac134 [file web_material_uhac134.zip › Supplement Fig. S4.jpg]

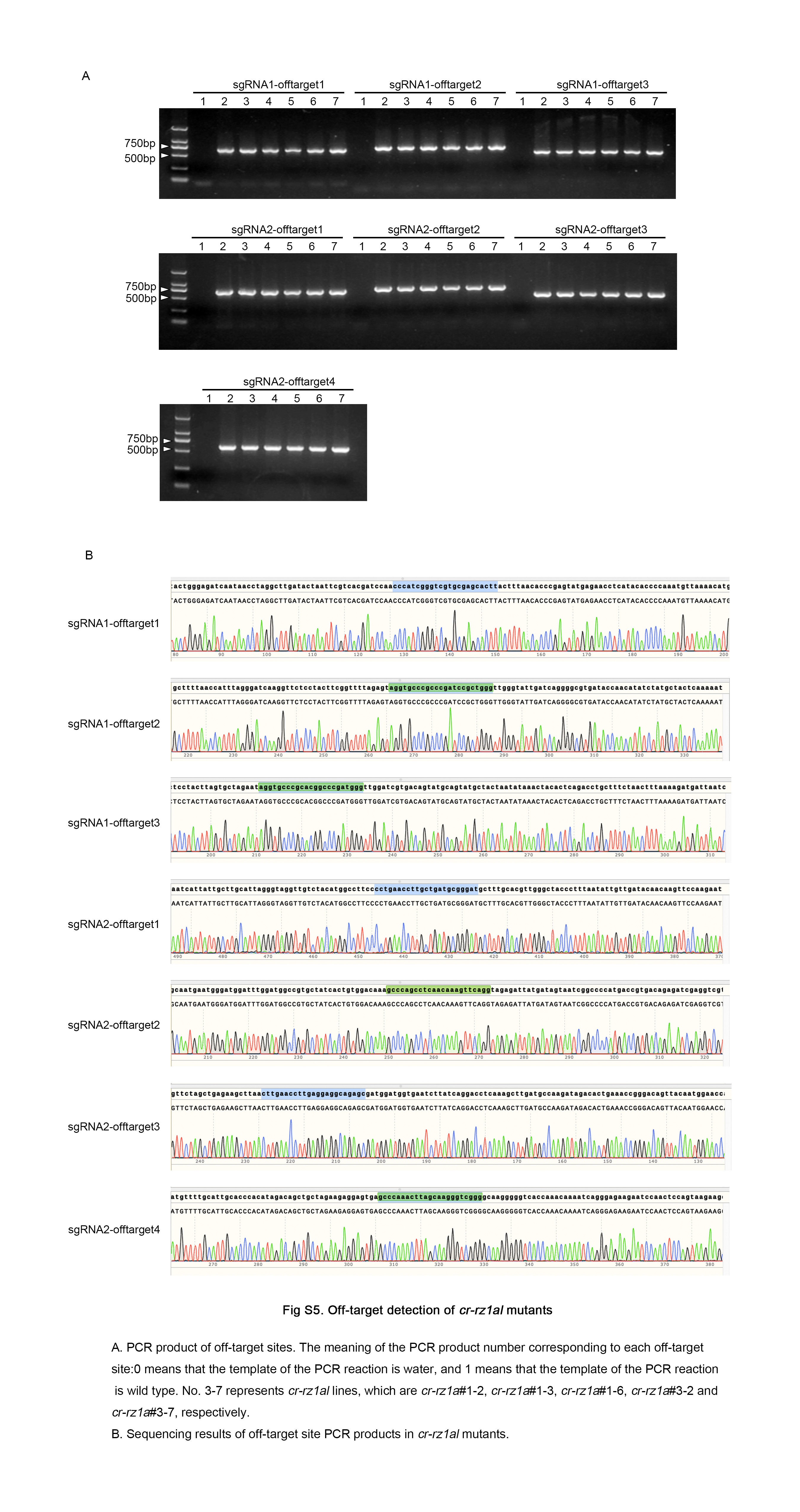

Supplement: Web_Material_uhac134 [file web_material_uhac134.zip › Supplement Fig. S5.jpg]

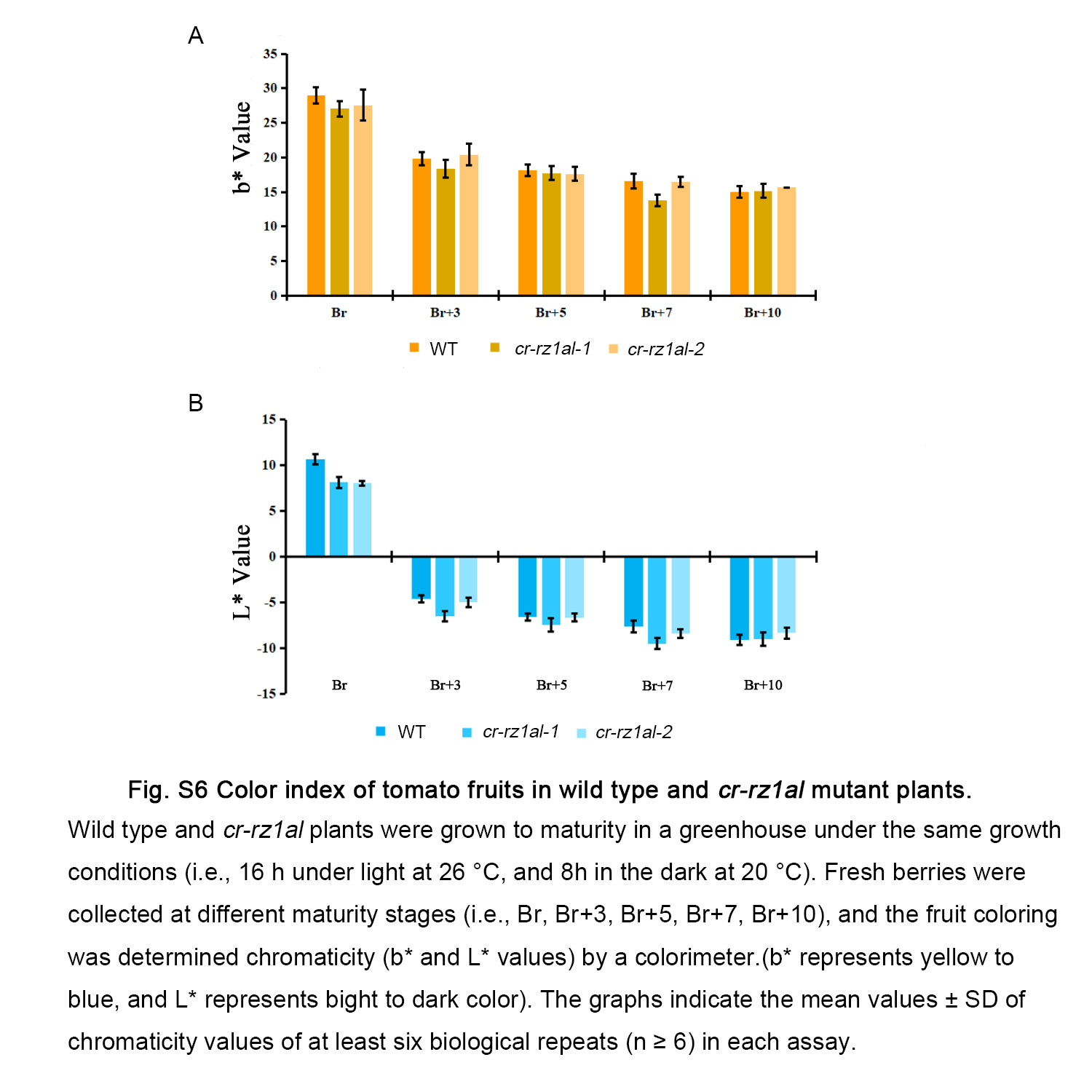

Supplement: Web_Material_uhac134 [file web_material_uhac134.zip › Supplement Fig. S6.jpg]

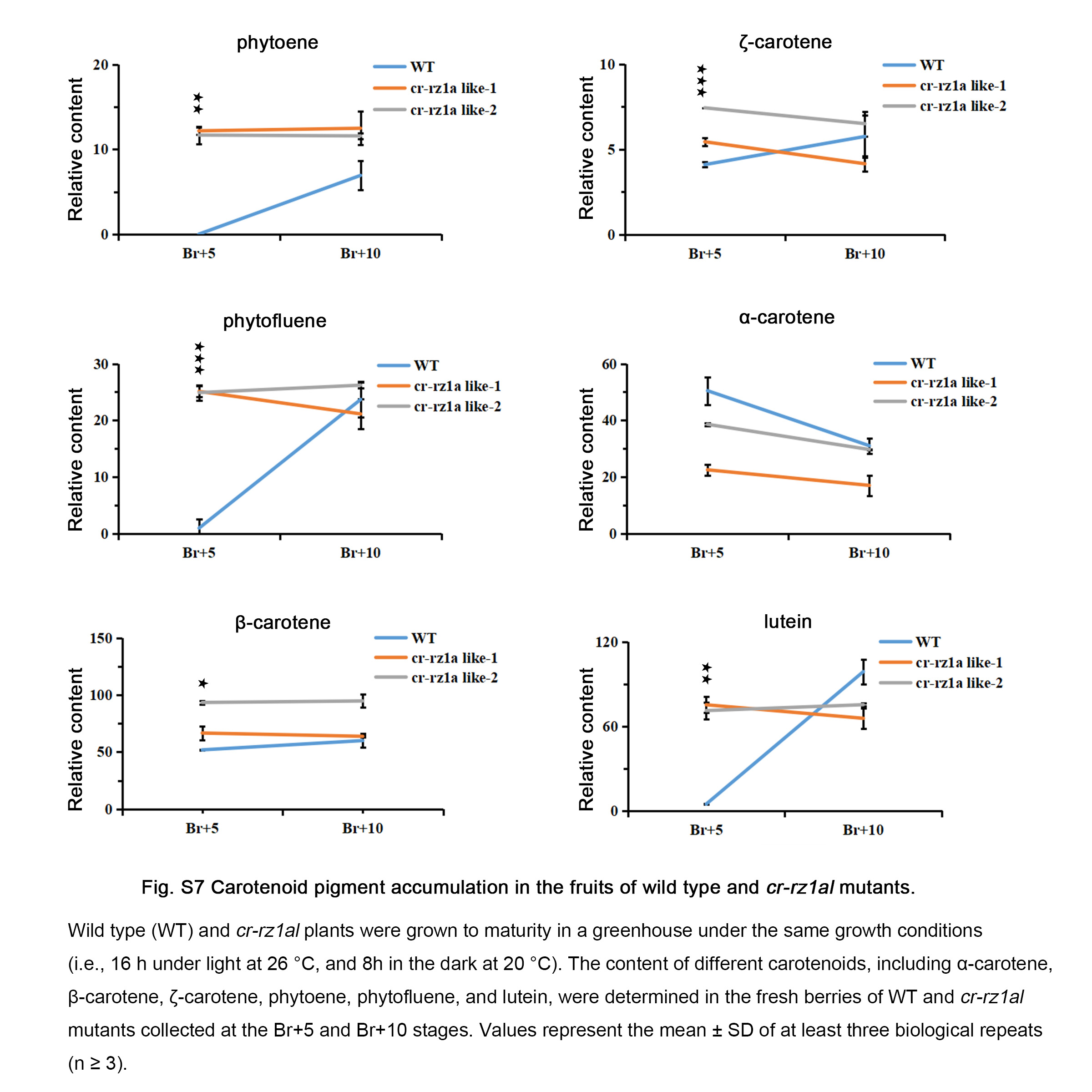

Supplement: Web_Material_uhac134 [file web_material_uhac134.zip › Supplement Fig. S7.jpg]

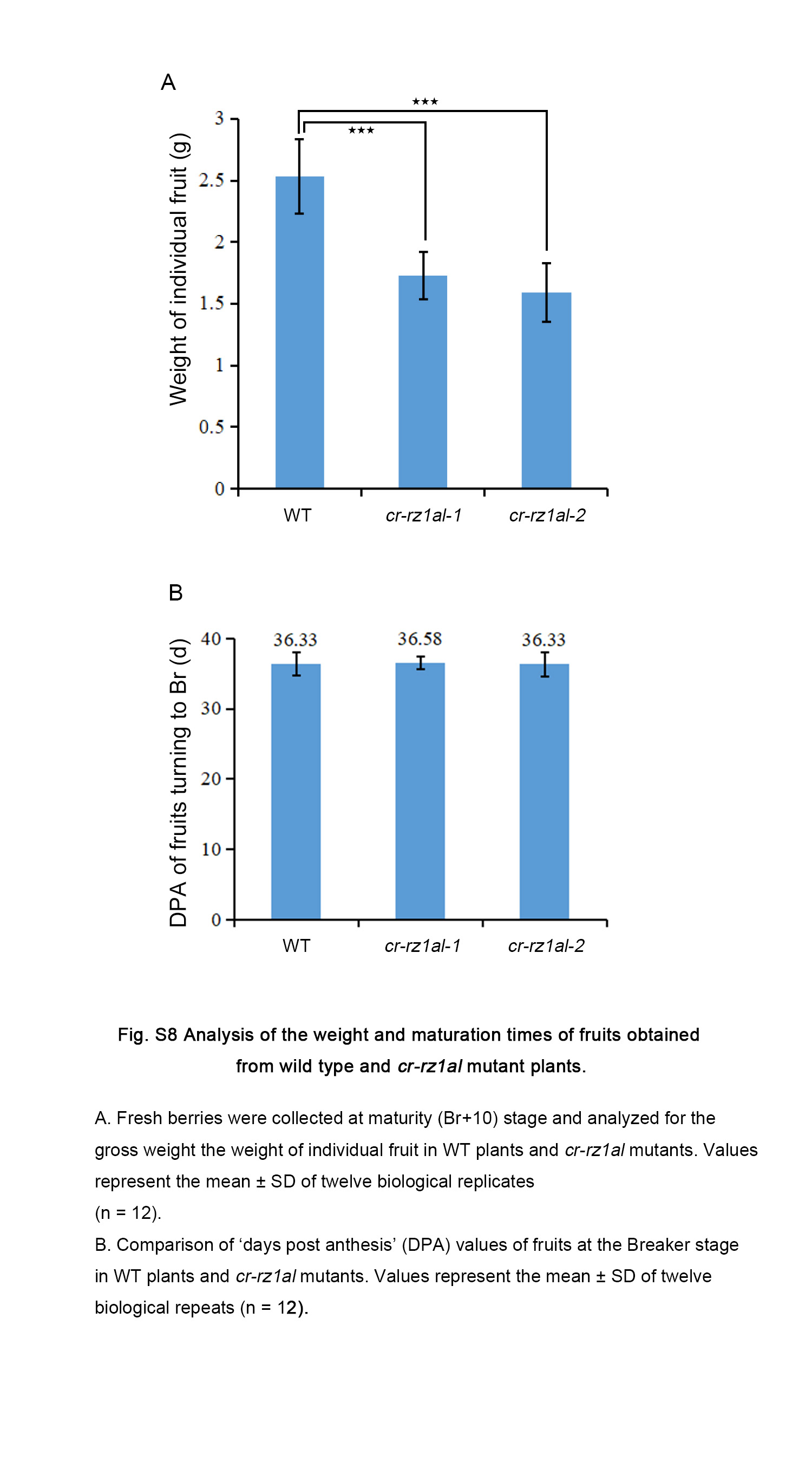

Supplement: Web_Material_uhac134 [file web_material_uhac134.zip › Supplement Fig. S8.jpg]

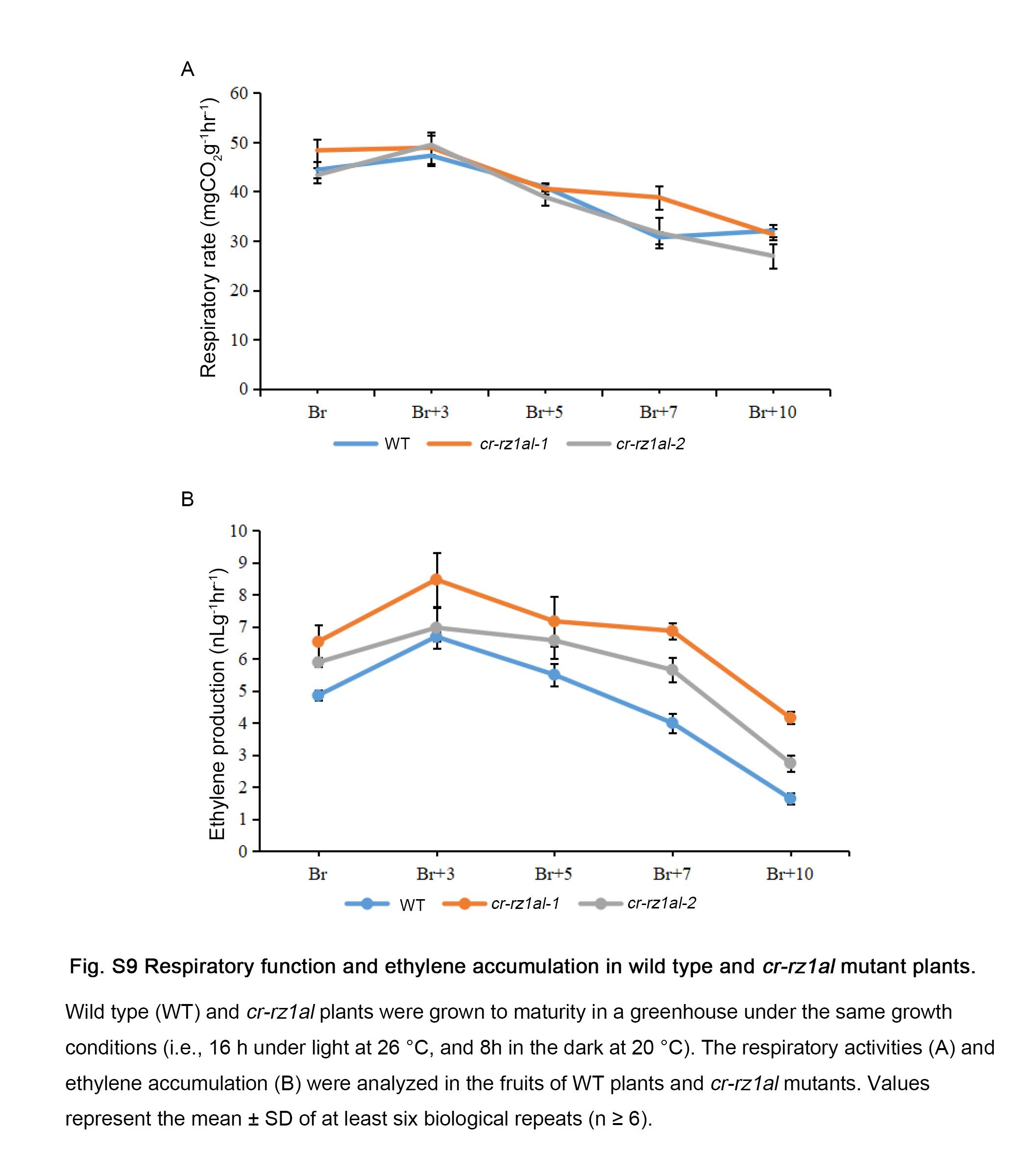

Supplement: Web_Material_uhac134 [file web_material_uhac134.zip › Supplement Fig. S9.jpg]
